# Fabrication of Living Entangled Network Composites Enabled by Mycelium

**DOI:** 10.1002/advs.202309370

**Published:** 2024-03-13

**Authors:** Hao Wang, Jie Tao, Zhangyu Wu, Kathrin Weiland, Zuankai Wang, Kunal Masania, Bin Wang

**Affiliations:** ^1^ Department of Mechanical Engineering City University of Hong Kong Kowloon Hong Kong; ^2^ Shaping Matter Lab Faculty of Aerospace Engineering Delft University of Technology Delft 2629 HS Netherlands; ^3^ School of Materials Science and Technology Nanjing University of Aeronautics and Astronautics Nanjing Jiangsu 211106 China; ^4^ School of Materials Science and Engineering Southeast University Nanjing 211189 China; ^5^ Department of Mechanical Engineering The Hong Kong Polytechnic University Hung Hom Kowloon Hong Kong

**Keywords:** living composites, mechanical properties, mycelium, phase separation

## Abstract

Organic polymer‐based composite materials with favorable mechanical performance and functionalities are keystones to various modern industries; however, the environmental pollution stemming from their processing poses a great challenge. In this study, by finding an autonomous phase separating ability of fungal mycelium, a new material fabrication approach is introduced that leverages such biological metabolism‐driven, mycelial growth‐induced phase separation to bypass high‐energy cost and labor‐intensive synthetic methods. The resulting self‐regenerative composites, featuring an entangled network structure of mycelium and assembled organic polymers, exhibit remarkable self‐healing properties, being capable of reversing complete separation and restoring ≈90% of the original strength. These composites further show exceptional mechanical strength, with a high specific strength of 8.15 MPa g.cm^−3^, and low water absorption properties (≈33% after 15 days of immersion). This approach spearheads the development of state‐of‐the‐art living composites, which directly utilize bioactive materials to “self‐grow” into materials endowed with exceptional mechanical and functional properties.

## Graphical abstract

A new material fabrication approach is introduced that leverages biological metabolism‐driven, mycelial growth‐induced phase separation to bypass high‐energy cost and labor‐intensive synthetic methods. This approach spearheads the development of state‐of‐the‐art living composite materials, seamlessly integrating ″self‐growth″ into materials endowed with exceptional mechanical and functional properties.

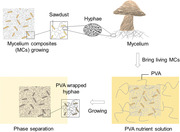

## 1 Introduction

Organic polymer‐based composite materials are ubiquitously providing functions and properties in various modern industries. However, their current production processes usually feature high energy consumption and noxious gas emissions, causing aggravating environmental impacts that need to be addressed by developing new fabrication technologies.^[^ ^1^, ^2^, ^3^ ^]^ Natural biomaterials are highly efficient in low energy‐cost synthesis of eco‐friendly materials with superior mechanical and functional properties. For instance, biological metabolism‐driven self‐assembly organizes basic building blocks into complex hierarchical structures with precise morphological control, self‐healing capabilities, and responsiveness to environmental stimuli.^[^ ^4^, ^5^, ^6^, ^7^, ^8^, ^9^, ^10^ ^]^ These characteristics offer a promising design paradigm for developing sustainable smart materials. For example, Balasubramanian et al. employed algae to fabricate regenerative photosynthetic materials with potential applications in artificial leaves, photosynthetic bio‐garments, and adhesive labels.^[^ ^7^ ^]^ Drawing inspiration from living organisms and reconfiguring the fundamental units into structurally and functionally sophisticated materials can provide an effective strategy for creating sustainable and intelligent materials, thereby providing new solutions to addressing the environmental issues associated with the conventional manufacturing of organic polymer composite materials.

**Figure 1:**
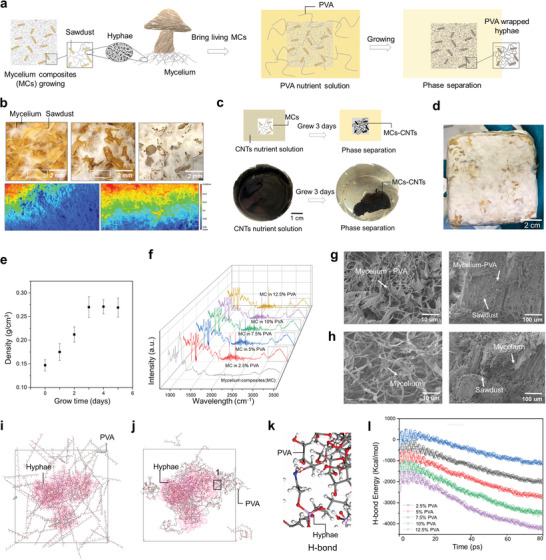
Biofabrication of self‐regenerative entangled network composites through living mycelium and phase separation. a) The fungal mycelium is a fibrous network consisting of interwoven, microscale filaments (fungal cells being called hyphae). Mycelium composites (MCs) are introduced into a PVA‐containing liquid nutrient medium for cultivation. During cultivation, the growing hyphae not only form the fibrous network connecting sawdust flakes but also autonomously induce phase separation to assemble the PVA, leading to the grown/fabricated mycelium‐PVA composites (MPCs). b) The macroscale morphologies of growing MCs over time from the left to the right: 4, 7, and 12 days (captured by a digital camera). 3D optical microscopic images enlarge the zones. Colors indicate the heights. c) The MCs initiate phase separation in the carbon nanotubes (CNTs) solution. MC is immersed in the CNTs solution, and after 3 days of growth, phase separation of CNTs from the remaining solution occurs, leading to the adsorption of CNTs onto the MCs. Consequently, the solution reaches the appearance of a clear solution without CNTs. d) Digital photo of the MPC. e) Relationship between growth time and density of the MPC. f) Fourier transform infrared (FTIR) spectra of the MCs in media with different mass fractions of PVA. g) Microstructure of the MPCs: SEM images of the cross‐section showing the mycelium and sawdust, and the mycelium having entangled hyphae covered by PVA. h) The SEM images of the cross sections of MCs. MD simulations result: i) The initial model of MCs‐10% PVA. j) The stable conformation after dynamic optimization. k) Enlarged view of region 1 in (j). H‐bond formed between hyphae and PVA. l) H‐bond energy change during the simulation.

Specifically, mycelium constitutes the vegetative, root‐like structure of a fungus, and is a network formed by interwoven micrometer‐sized filaments (**Figure** **1**a). These filaments, known as hyphae, are tubular fungal cells.^[^ ^11^ ^]^ Mycelium composites are typically composed of mycelium and natural sources (lignin reinforcements), and offer distinct advantages, including low energy costs, high sustainability, and biodegradability which contribute to a circular economy.^[^ ^12^, ^13^ ^]^ They are drawing increasing interest from research and commercial sectors for various applications such as construction, packaging, furniture, and insulation industries.^[^ ^14^ ^–,^ ^23^ ^]^ However, most of the fungal mycelium composites reported thus far remain in an inactive state, constraining their functional performance. Exploring novel functionalities by using living fungal activities shows great potential for creating new materials.^[^ ^24^, ^25^, ^26^, ^27^ ^]^ For instance, Gantenbein et al.^[^ ^24^ ^]^ ingeniously incorporated living mycelial cells into a gel for 3D printing regenerative, self‐cleaning robotic skin. McBee et al.^[^ ^25^ ^]^ pioneered in developing biocomposite blocks comprised of bacteria, fungi, and feedstock that can be assembled into human‐sized, living structures with self‐healing and environmental sensing capabilities.

In this work, we find the phase‐separating ability of growing mycelium and leverage this to assemble external macromolecules/nanoparticles to develop self‐regenerative composites with an entangled network structure (Figure 1a). To demonstrate the efficacy of our approach, we prepare a mycelial nutrient suspension containing 10 wt% polyvinyl alcohol (PVA)^[^ ^28^ ^]^ and introduce living mycelial composite materials (MCs) into the suspension, facilitating the phase separation of PVA from water. This process yielded self‐healing mycelium‐PVA composites (MPCs) exhibiting exceptional mechanical properties and resistance to water absorption. Importantly, this method significantly mitigates noxious gas emissions and reduces energy consumption associated with the processing of hybrid polymer composite materials in traditional mechanical blending techniques. Furthermore, we establish a multiscale analytical model of the hierarchical structure integrating molecular dynamics (MD) simulation^[^ ^29^ ^]^ and finite element analysis (FEA) simulation^[^ ^30^ ^]^ methods to unveil the mechanisms that govern mechanical failure and water absorption resistance.

## 2 Results and Discussion

### 2.1 Biofabrication Through Living Mycelium and Phase Separation

We introduce the type of *Ganoderma lucidum* fungi into sawdust (having a size range of 3–7 mm) (Figure S6, Supporting Information) and cultivate them to form the mycelium composites (MCs). The growth process of the MCs is depicted in Figure 1b. With increasing time, mycelial hyphae grow to connect the sawdust; then the mycelium becomes thicker after seven days and fills the gaps between the sawdust to form a bulk material. As can be observed in Figure 1b, the fungal growth pattern involves the extension of hyphae, which originates from the sawdust substrate and eventually develop into a network structure; such a hyphal network becomes denser over time.

We place the MCs into a nutrient solution containing 5% carbon nanotubes (CNTs) and observe the growth of mycelium (Figure 1c). Interestingly, the mycelium is able to attach and assemble CNTs, thereby covering the surface of the mycelium (Figure 1c; Figure S7, Supporting Information). It is noteworthy that inactivated mycelium lacks the capacity to induce this phenomenon (Figure S8, Supporting Information). This means that mycelium activity can be used to separate the phases (macromolecules/nanoparticles from solvent/water) and assemble the macromolecules and nanoparticles, enabling the development of more advanced and environment‐friendly composite materials. In a parallel manner, the MCs are introduced into a liquid nutrient solution containing 10 wt.% polyvinyl alcohol (PVA), allowed to grow for 3 days, and subsequently removed, being left in the air for additional 3 days. The resulting mycelium‐PVA composite materials (MPCs) are presented in Figure 1d. Through the assessment of density changes in the sample after water removal (Figure 1e), it is evident that following 3 days of MCs growth, a dynamic equilibrium is reached, and the trend of density increase diminishes, approaching 0.27 g cm^−^^3^. The Fourier‐transform infrared (FTIR) spectra results reveal that in comparison to the MCs, prominent peaks associated with hydrogen bonding in the range of 3000–3500 cm⁻¹ show up for the MPCs (Figure 1f).

Microstructural analyses show that the living mycelium assembles PVA onto the growing hyphae and attains a concurrent full coverage of PVA on variously sized hyphae, hyphal hinges, and hypha‐sawdust joints. For MPCs (Figure 1g), the microstructural features, including the mycelium composed of interwoven filamentous hyphae and the sawdust, resemble those of MCs, except that the hyphae show larger diameters than MCs (Figure 1h) due to the presence of PVA on the hyphal surface and the assembled PVA fills spaces at hyphal hinges and between hyphae‐sawdust joints (Figure 1g). FTIR analysis (Figure S2, Supporting Information) discloses the presence of hydroxyl, carboxyl, and amide functionalities in *Ganoderma lucidum*. This observation is ascribed to the composition of polysaccharides (≈60%), lipids (10%), and proteins (≈30%) comprising the fungal cell wall. Molecular dynamics (MD) simulation is employed to observe the behavior of phase separation. This simplified molecular model is based on the chemical composition while overlooking several factors such as molecular weight, secondary and tertiary structures, and arrangements of phases. Further work will focus on developing more accurate molecular models to include these considerations. The composition encompasses all functional groups available for intermolecular interactions. The initial model of MPCs is shown in Figure 1i. After dynamics optimization, the system reaches a dynamic equilibrium. It can be seen that PVA molecules are adsorbed and entangled on the surface of the hyphae (Figure 1j,k). The adsorption driving force is mainly the formation of hydrogen bonds (Figure 1l).

### 2.2 Self‐Healing Properties

**Figure 2:**
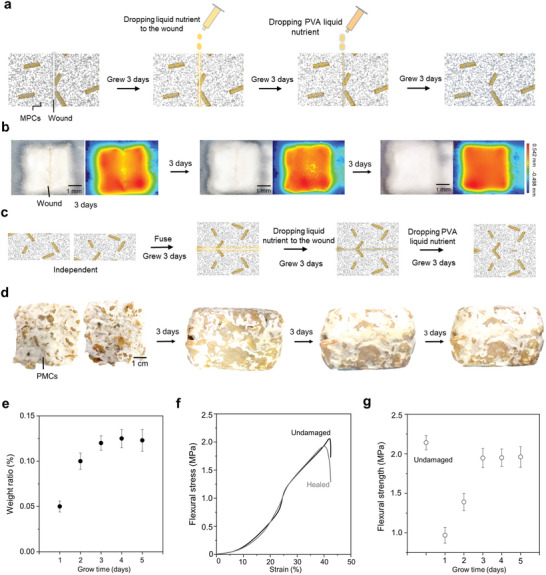
Self‐healing of MPCs. For small‐scale damages: a) The healing experiment, a 1 cm‐long and 1 mm‐deep cut is made on the surface of a 2 cm × 2 cm × 1.5 cm sample. Following the incision, the sample is allowed to stand at room temperature for 3 days. Then, the liquid nutrient solution is introduced to the wound. After 3 days of growth, a nutrient solution containing 10% PVA is applied dropwise and continued to cultivate the sample for additional 3 days. b) After 3 days of growth, the wound is filled by developing mycelium; after 3 days of growth, the wound is completely healed. Colored images are 3D optical images showing the height distributions. For large damages: c) Two independent MPCs are stacked together. After 3 days of growth, the liquid nutrient solution is added dropwise. After 3 days of growth, a nutrient solution containing 10% PVA is added dropwise and the growth continues for 3 days. d) Two separate samples (observed from the top view) are allowed to grow independently. Subsequently, two samples are vertically stacked and grown for 3 days (observed from the side view), resulting in the connection of the wound by mycelium. The two stacked samples are grown for an additional 3 days after the addition of liquid nutrient solution (observed from the side view), showing the initial suturing of the wound. After adding drops of PVA nutrient solution and growing for 3 days, they fuse into a single bulk material through the process of self‐healing by phase separation. e) The weight change (in ratio, weight change over initial weight) overgrowth time. f) The typical flexural stress‐strain curves. g) The flexural strength variation of the sample bearing the cut damage during the self‐healing stage.

For self‐healing of small‐scale damages, the repair method is illustrated in **Figure** **2**a. Micro‐scratches are deliberately made on the upper surface of the MPCs (1 cm long × 1 mm deep on a 2 cm × 2 cm × 1.5 cm sample). After allowing it to stand at room temperature for three days, 1–2 mL liquid nutrient solution is added to the wound. Following three days of growth, an extra 1–2 mL 10% PVA liquid nutrient solution is added dropwise. The living mycelium induces phase separation of the PVA solution, causing the PVA to envelop the hyphae. It is evident that, after nurturing the MPCs wound with dropping nutrient solution for three days, the wound is initially repaired by mycelium (Figure 2b). Subsequently, after three days of growth with the dropwise addition of PVA nutrient solution, the wound is completely repaired. For large‐scale repairs, the healing process is depicted in Figure 2c. Two separate MPCs with dimensions of 5.5 cm × 5.5 cm × 2.5 cm are stacked together for three days of growth. Then liquid nutrient solution is supplied to the gap between the superimposed MPCs for additional three days of growth. Following this, PVA nutrient solution is added dropwise, and the gap is filled with mycelium after growth of three days (Figure 2d). It can be observed that after the nutrient solution is added drop‐by‐drop for three days, the wound is essentially sutured, and following extra three days of growth with the PVA nutrient solution, the wound is healed, and large‐sized MPCs are formed.

Three‐point bending tests are applied to characterize the repair efficiency of the sample during the phase separation stage, and the results are illustrated in Figure 2f,g. Undamaged MPCs samples typically exhibit a flexural strength reaching a plateau of ≈2.2 MPa. In comparison, the cut‐damaged sample displays a strength of ≈1.96 MPa after healing for three days, which is close to 90% of the original strength. The strength essentially reaches dynamic equilibrium after the third day (Figure 2g). This high self‐healing efficacy validates that this method can be used to sustainably manufacture self‐regenerative composites at large scales.

### 2.3 Compressive Mechanical Properties

**Figure 3:**
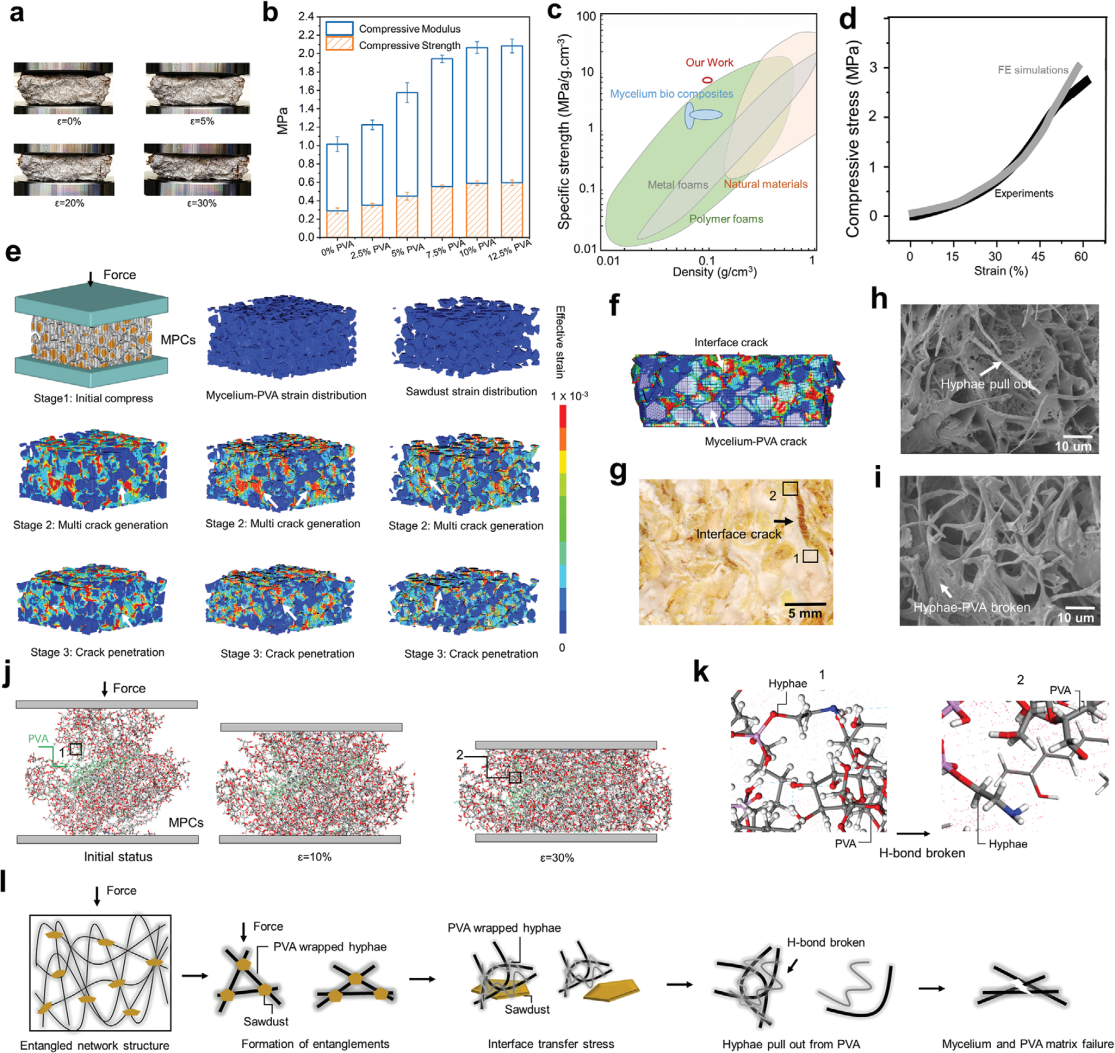
Compressive mechanical behavior of MPCs. a) Compression testing of the MPCs. b) Compressive strength and modulus. c) The Ashby plot of specific strength versus density, comparing the MPCs with other relevant MCs and other cellular solids. d) Compressive stress–strain curves from experiments and finite element analysis (FEA) simulation. e) FEA simulating the compressive deformation processes. f) The damage and failure pattern from FEA simulation. g) The cross‐sectional photo. h) The magnified SEM image of position 1 in (g). i) The magnified SEM image of position 2 in (g). j) Molecular dynamics (MD) compression simulation results. k) The magnified image of positions 1 and 2 in (j). l) Compressive strength enhancement mechanism. The intertwined network structure consists of the mycelium with PVA‐wrapped hyphae and the sawdust flakes, both forming resilient entangled triangular structures. As compression proceeds, the network entanglements deform and sustain load, and strain concentrations gradually emerge at numerous interfaces of the many hyphae‐PVA‐sawdust entanglements; these microscale mechanisms effectively distribute damages over large volumes and delay the final failure. Moreover, the hydrogen bonding formed between the hyphae and PVA makes the pull‐out and fracture of the hyphae from the PVA matrix to consume more load, ultimately giving rise to enhanced fracture stress levels of both the mycelium and the PVA matrix.

Inducing phase separation through living mycelium effectively produces cellular composites with exceptional mechanical strength. **Figure** **3**a demonstrates the compressive deformation (strain ranging from 0% to 30%) of MPCs, in which the sample densifies, and microcracks gradually emerge. The compressive strength of MPCs reaches 2.2 MPa (Figure 3b), markedly superior to that of existing mycelium composites and mycelium composites reinforced with additional materials reported in the literature (Table S1, Supporting Information). Considering the density (0.27 g cm^−3^ for MPCs in this study), the specific compressive strength is also significantly higher than that of various natural organism‐based composites (Table S1, Supporting Information). This superior compressive strength and lightweight feature become more apparent when compared with different material types, as illustrated in the Ashby map (Figure 3c). Despite being comprised of organic polymer‐based biological mycelium, sawdust, and PVA, which inherently have lower mechanical strength than polymeric cellulose and metals, the fabricated MPCs exhibit specific strength properties that surpass the upper bounds of polymer foams (the highest specific strengths). This breakthrough expands the current property range of polymer foams by introducing stronger and lighter polymeric cellular composites.

The remarkable mechanical properties of MPCs are analyzed through multi‐scale simulations that reveal phase separation‐induced strengthening mechanisms. The finite element analysis (FEA) produces compressive stress‐strain curve behaviors that agree well with experiments (Figure 3d), validating results from the simulations. Under compressive loading, the initial load is sustained by the entangled network structure formed by PVA‐wrapped mycelium and sawdust (Figure 3e). As compression continues, the entangled network structure deforms, facilitating the transfer of the load to the PVA‐mycelium and sawdust. Cracks initiate and propagate at the interface, resulting in interface failure. Subsequently, the PVA‐mycelium matrix and sawdust undertake the load‐bearing role (Figure 3f). A comparison of MPCs failure modes (Figure 3f–i) reveals that cracks primarily occur at the interface between PVA‐mycelium and sawdust. Additionally, there are instances of hyphae pull‐out from the PVA and breakage, alongside failures observed in both the PVA and mycelium matrix. Microstructural observations (Figures 1g,h and 3h,i) vividly display the full coverage of assembled PVA on hyphal skeletons and hinges, a phenomenon explained through molecular analysis illustrating PVA molecules wrapping around hyphae (Figure 1j). In the molecular dynamics (MD) compression process, the destruction of hydrogen bonds between PVA and hyphae becomes apparent (Figure 3j). The robust PVA‐hyphae bonding is evidenced by intact, PVA‐bearing hyphae, showing no slippage or delamination at the PVA‐hyphae interface under compression deformations.

Therefore, the compression strength enhancement mechanism is elucidated as presented in Figure 3l. Throughout the compression process, the load is effectively sustained by the intricately intertwined network structure. The mycelium, entangled with PVA and sawdust, forms a resilient triangular‐like entanglement structure. As compression progresses, the deformation of this entanglement structure disperses the load, and stress/strain concentrations gradually occur at numerous interfaces of the hyphae‐PVA‐sawdust entanglements. These microscale deformation mechanisms effectively delocalize damages and delay the final failure. This process persists until the mycelium‐sawdust interface undergoes dissociation. Furthermore, the hydrogen bond formation between mycelium and PVA plays a vital role in the pull‐out and fracture of hyphae from the PVA matrix. This nanoscale mechanism efficiently dissipates the load, leading to the fracture of both the mycelium and the PVA matrix at higher stress levels.

### 2.4 Water‐Absorption Resistance and Self‐Regenerative Properties

**Figure 4:**
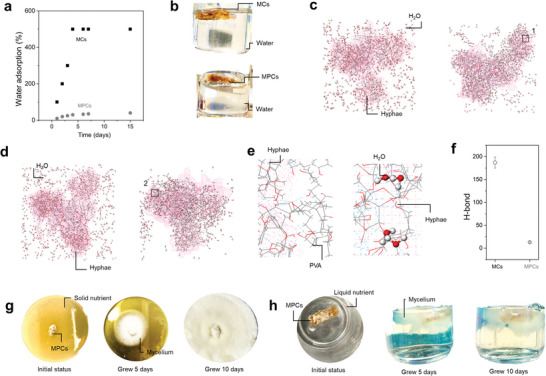
The water absorption properties and self‐regenerative properties. a) The weight gain of absorbed water with increasing immersing time for the MPCs and MCs. b) Immersing the MCs sample and the MPCs sample (sample size 20 mm × 5 mm × 2 mm) in water for 72 h. The MPCs remains buoyant. MD simulations results of c) MPCs. d) MCs. e) The enlarged image of positions 1 and 2 in (c) and (d), respectively. f) The number of hydrogen bonds. The growth process of MPCs (after 100 days storage at room temperature) placed in solid g) and liquid media h).

The MPCs obtained by living MCs‐induced PVA phase separation have significantly low water absorption (**Figure** **4**a). Figure 4b shows that when immersed in water, the MCs sample gradually sinks into water, but the MPCs remain buoyant after 72 h. Conventional MCs easily absorb a large amount of water due to the hydrophilic nature of the lignocellulosic constituent and the abundant pores within the material, thus sinking into the water despite a smaller density than water. This is one major drawback that limits the wide application of mycelium‐based composites. The water absorption results highlight that the MPCs have substantially lower water absorptions (35%) than the MCs (≈486%); this water absorption is better than other mycelium composites reported in the literature (Table S2, Supporting Information). In addition, the superior low water absorption ability stabilizes when the PVA content in the cultivating medium is 10%, consistent with the trend of PVA content affecting mechanical properties. This represents a new effective approach to address issues of water absorption‐related failures and enhance water‐resistant properties of composites composed of fungal and lignin.

The mechanisms for such noteworthy water‐absorption properties rely on the assembly of PVA occupying hydrogen‐bond formable molecular sites in the hyphae. For the MCs, water molecules easily form a large amount of hydrogen bonds with the polysaccharides in the hyphae and sawdust, as shown in Figure 4e. However, the MPCs form hydrogen bonds between the PVA molecules and the polysaccharides in the hyphae and sawdust of the MCs. This leads to a decreased number of available sites in hyphae and sawdust to constitute hydrogen bonds with water molecules, ultimately leaving water molecules in free form. The number of hydrogen bonds of water molecules in the MPCs is smaller than the MCs (Figure 4f), further suggesting the reduced number of hydrogen‐bond formable molecular sites due to consumption by assembled PVA molecules. Such hydrogen‐bond dominated mechanism of MPCs simultaneously attains synergistic high mechanical strength and low water absorption property, which are usually conflicting properties in current mycelium composites (higher content of reinforcing lignocellulosic filler/reinforcement increases strength but decreases anti‐water absorption property).

We cultured MPC samples that had been sealed and stored at room temperature for 100 days in both solid and liquid media, as depicted in Figure 4g,h. Impressively, new mycelium emerged after ten days of growth, providing further evidence of the material's robust regenerative capabilities.

## 3 Summary and Outlook

In this work, we develop new self‐regenerative composite materials featuring an entangled network structure by inducing phase separation through living mycelium, utilizing biological metabolism‐driven hyphal growth rather than traditional synthesis or mechanical mixing methods. The fabricated composites can self‐heal large damages and show exceptional mechanical (specific strength of 8.15 MPa g.cm^−3^) and low water absorption (≈33% water absorption after 15 days immersion) properties, which will broaden the application of mycelium composites to encompass load‐bearing and functional territories. It also exhibits a robust regenerative capacity, as samples left for 100 days can still generate new mycelium. We reveal the strengthening and water‐resistant mechanisms through experiments and multi‐scale simulations to show that mycelial hyphae and hyphae‐sawdust interfaces are strengthened by the full, tight coverage of the reinforcing PVA on mycelium and sawdust through hydrogen bonds. When exposed to external loads, the network interlocking structure created by PVA‐encased mycelium and sawdust supports and absorbs the applied load.

Thus, we bring forth a new composite material fabrication approach that i) manufactures materials simultaneously attaining self‐healing, high strength, and water resistance, ii) is low‐cost and high‐efficacy through directly using the natural evolution refined growing and bulk‐material forming features of fungal mycelium; and iii) is versatile to produce diverse functionalities by changing/diversifying the phase separation of heterogeneous materials induced by the mycelial hyphae. Future work is directed to extend this mycelium‐assisted fabrication approach to creating novel high‐performance, multifunctional composite materials supported by in‐depth analysis and more advanced modeling.

## 4 Experimental Section

The Experimental Section is available in the Supporting Information.

## Conflict of Interest

The authors declare no conflict of interest.

## Author Contributions

H.W. contributed to the conceptualization. H.W., J.T., Z.K.W., K.W., K.M., and B.W. worked on the methodology. H.W. and Z.Y.W. developed the software. H.W., Z.Y.W., J.T., K.M., Z.K.W., K.W., and B.W. performed the investigation. J.T., K.M., and B.W. performed the supervision. H.W. wrote the original draft. T.J., K.M., Z.Y.W., K.W., Z.K.W., K.M., and B.W. wrote the original draft and reviewed and edited the final manuscript.

## Supporting information

Supporting Information

## Data Availability

Data sharing is not applicable to this article as no new data were created or analyzed in this study.
