# Supplementary material for: Fabrication of Living Entangled Network Composites Enabled by Mycelium
Source: Adv Sci (Weinh). 2024 Mar 13;11(24):2309370. doi: 10.1002/advs.202309370 (PMC11200020; doi:10.1002/advs.202309370)
Supplement: Supplementary file 1 — Supporting Information [file ADVS-11-2309370-s001.pdf]

ADVANCED SCIENCE
Open Access

Supporting Information

for *Adv. Sci.*, DOI 10.1002/advs.202309370

Fabrication of Living Entangled Network Composites Enabled by Mycelium

Hao Wang, Jie Tao, Zhangyu Wu, Kathrin Weiland, Zuankai Wang*, Kunal Masania* and Bin Wang**

Supporting information

Fabrication of living entangled network composites enabled by mycelium

Hao Wang[1,2], Jie Tao[3]*, Zhangyu Wu[4], Kathrin Weiland[2], Zuankai Wang[5]*, Kunal Masania[2]*, Bin Wang[1]*

[1] Department of Mechanical Engineering, City University of Hong Kong, Hong Kong

[2] Shaping Matter Lab, Faculty of Aerospace Engineering, Delft University of Technology, Delft, 2629 HS, Netherlands

[3] School of Materials Science and Technology, Nanjing University of Aeronautics and Astronautics, Nanjing, Jiangsu, 211106, China

[4] Jiangsu Key Laboratory of Construction Materials, School of Materials Science and Engineering, Southeast University, Nanjing, Jiangsu, 211189, China

[5] Department of Mechanical Engineering, The Hong Kong Polytechnic University, Hong Kong

*Corresponding Authors: taojie@nuaa.edu.cn; k.masania@tudelft.nl; zk.wang@polyu.edu.hk; bwang55@cityu.edu.hk

Methods

Materials

The following chemicals were purchased and used: malt extract (Sigma-Aldrich), peptone (Sigma-Aldrich), agar (Sigma-Aldrich), D-glucose (Sigma-Aldrich), yeast extract (Sigma-Aldrich), $NaC_5H_8NO_4$ (Sigma-Aldrich), KH_2PO_4 (Sigma-Aldrich), K_2HPO_4 (Sigma-Aldrich), and $MgSO_4$ (Sigma-Aldrich), Polyvinyl alcohol (PVA, Sigma-Aldrich), and Carbon nanotubes (CNTs, Nanjing XF Nanotechnology Co., Ltd.). The fungal specie of Ganoderma lucidum was employed to culture. The sawdust was obtained from poplar wood.

Mycelium growth nutrition

Solid culture medium: Ganoderma lucidum was kept in Petri dishes containing malt agar. Malt Agar contains agar (15 g), peptone (10 g), and malt extract (30 g), dissolved in deionized water (1 L). Liquid culture medium: D-glucose (15 g), peptone (2.5 g), yeast extract (3 g), KH_2PO_4 (1 g), K_2HPO_4 (0.2 g,) and $MgSO_4$ (0.5 g) were dissolved in 1 L of deionized water and adjusted to pH 5.5. The mixture was autoclaved (121 °C, 1 h).

Preparation of mycelium composites

The poplar sawdust, sized between 3-7mm, was soaked in water for 24 hours and then sterilized by autoclaving at 121°C for 1 hour. Agar, used for the cultivation of mycelium, was cut into several pieces and mixed with the sterilized sawdust. The mixture was placed in a plastic bag, shaken thoroughly, and grown under aseptic and aerobic conditions for 7 days. After a period of 7 days, the blend of mycelium and sawdust was thoroughly crushed inside a plastic bag, vigorously shaken, and subsequently transferred into a mold with dimensions of 55 x 55 x 30 mm^3. The mixture was subsequently allowed to undergo further growth for an additional 7 days, leading to the formation of a solid block material. The block was then inverted and subjected to an additional 3 days of growth to obtain the final mycelium composite product.

For the preparation of PVA samples assembled using living mycelia, we followed the procedure outlined in the above for mycelia culture. PVA was introduced into the culture medium with mass fractions ranging from 0% to 12.5% at 2.5% intervals. As for the assembled carbon nanotubes (CNTs) samples, 5% CNTs were added to the nutrient solution. Before

autoclaving, these mixtures underwent ultrasonic stirring for homogenization to disperse the additives. Regarding mycelial composites assembled via the PVA nutrient solution, live mycelial composites prepared in above were positioned in 1L flasks containing 600 ml of medium. The flasks were continuously shaken for 7 days at 50% RH and 23°C at 120 rpm. To obtain inactivated samples, the specimen was placed in an oven at 65°C for 24 hours.

Material Performance Testing and Characterization

Morphology and composition Analysis. Digital images were captured using an iPhone XS. Surface morphology was characterized using a field-emission scanning electron microscope (F100, Japan). The morphologies showing the height differences are observed using a Keyence VR-5000 wide-area 3D measurement system (Keyence International, Mechelen, Belgium) to generate 3D optical microscopic images.

The surface of the samples was analysed by acquiring FTIR spectra using a Spectrum Two IR spectrometer (Perkin Elmer). The spectra covered a range from 4000 to 500 cm^{-1} with a resolution of 4 cm^{-1} and a total of 64 scans were performed. The test samples were maintained at room temperature. To ensure the reproducibility of the obtained spectra, three replicate samples were measured.

Particle size analysis. Sawdust flakes were well dispersed on a sheet of paper, and images were scanned at 600 dpi by a Canon Document Feeder (DADF-AP1, Canon, Inc., Tokyo, Japan). And convert binary images to a black background using ImageJ software (ImageJ 1.48 v, National Institutes of Health). Based on the best-fit ellipse, ImageJ analysed the basic geometric properties of the particles, including length, width, area, and perimeter. Aspect ratio, circularity, and circularity 2 were computed as form factors, and a minimum of 500 particles per sample were analysed.

Bending test. Three-point bending tests were conducted using a universal testing machine (Zwick - 10kN). The specimens, measuring 10 × 30 × 10 mm, were subjected to bending stress at a rate of 2 mm/min. Flexural property values were calculated as the average of five samples. The flexural strength (σ) was determined using the following equation:

$$Flextural\ strength = \frac{3Fs}{2wh^2} \tag{1}$$

where F is the applied load, s is the span, w is the width of the sample, and h is the

height of the sample.

Compression test. For the compression tests, each specimen underwent compression at a rate of 1 mm/min. All mechanical testing was performed using a Zwick-10kN tensile/compression machine. A minimum of five samples were tested for each condition.

Water absorption test. The inactivated mycelium composite materials with and without assembling PVA, having dimensions of 10 mm in length, 5 mm in width, and 5 mm in thickness, were immersed in a container filled with distilled water at room temperature. The weight was measured after 15 days to determine the amount of water absorbed.

Molecular dynamics simulation

The MCs are modeled and presented in **Figure S1**. By analyzing the FTIR results of Ganoderma lucidum (**Figure S2**), it is determined that polysaccharides (approximately 60%) constitute the main component, followed by proteins (around 30%) and lipids (about 10%). The main constituents of the hyphae cell wall are polysaccharides, comprising mannans, ß-glucans, and phospholipids, with mass ratios of 60%, 2%, 28%, and 10%, as shown in **Figure S1b**. The sawdust molecular model is primarily composed of lignin, cellulose, and hemicellulose, with mass ratios of 30%, 45%, and 15%, respectively, as depicted in **Figure S1c**. The optimized model of the mycelium sawdust composite materials is illustrated in **Figure S1d**. Single-chain PVA molecules are employed to represent the PVA model, as presented in **Figure S1e**.

Model Construction. FTIR analysis (**Figure S2**) unveils the presence of hydroxyl, carboxyl, and amide functionalities in Ganoderma lucidum. This observation is attributed to the composition of polysaccharides (approximately 60%), lipids (10%), and proteins (around 30%) constituting the fungal cell wall [1]. The molecular simulation in this context primarily pertains to the hyphae state when devoid of water. During the establishment of a molecular representative model of hyphae, emphasis is placed on considering functional groups that can depict interactions between molecules. Guided by the FTIR peak area ratios, a model for each molecular component is created, forming a hyphae reaction model. In this model, chitin emerges as the predominant component of polysaccharides, accounting for approximately 60%, while mannan and β-glucans represent the primary components of proteins, constituting about

30%. Phospholipid accounts for roughly 10%. β-glucans are constructed based on provided chemical formulas, with their monosaccharides uniformly being glucose [2]. Mannans exhibit a main chain consisting of mannose residues linked by α-(1→6) glycosidic bonds, and their side chains consist of multiple mannose molecules connected by α-(1→2) and α-(1→3) glycosidic bonds. As for chitin, its chemical structure, resembling cellulose, forms a polymer composed of hundreds of β-(1-4)-linked D-glucose units. The arrangement of components from the outer wall of the hyphae, as per reference literature [3, 4], facilitated the construction of a molecular model representing the hyphae.

We employed poplar sawdust in our study, presenting the FTIR spectra in **Figure S3**. The -OH tensile vibration peak is evident at 3390 cm^{-1}, and the band at 2907 cm^{-1} corresponds to the C-H tensile vibration of methylene in cellulose. The bending vibration peak of the C-H bond in cellulose and hemicellulose is observed at 1368 cm^{-1}. At 1626 cm^{-1}, the peak is associated with the benzene ring carbon skeleton vibration in lignin, indicating the presence of cellulose (approximately 45%), hemicellulose (about 15%), and lignin (about 30%) in poplar sawdust [5]. Molecular dynamics (MD) simulations are directed toward delineating intermolecular interactions, prioritizing the characterization of functional group types and content. Consequently, we constructed a molecular model comprising 45% cellulose molecules, 15% hemicellulose molecules, and 30% lignin molecules to represent sawdust. The molecular structure of cellulose encompasses a disordered non-fixed phase and an ordered crystal phase. We opted for the (1 1 0) surface to delineate the structure of the crystal cellulose surface, considering it as the primary exposed surface of crystal cellulose. Glucosolic acid is selected as the principal component of hemicellulose, with fragments consisting of 10 D-xylopyranosyl residues connected by linear (1→4)-β-links. Leveraging available experimental data on the average chemical composition of white poplars [6], we formulated a structural model for a single lignin molecule. Each molecule comprises 45 basic units, with a Guaiacyl /Syringyl ratio of 1.74 and a molecular weight of 9.5 kDa.

A simulation model of mycelium-sawdust composites (MCs) was constructed using the Monte Carlo algorithm, with a box size of 10 nm X 10 nm X 10 nm. The weight ratio between the two components was determined by calculating the ratio of their mass increase after a 10-

day growth period. Subsequently, structural optimization and dynamics calculations were carried out to obtain stable structures that represent the mycelium-sawdust composite material model. The box size was then expanded to 15 nm X 15 nm X 15 nm and filled with varying masses of PVA molecules around the MC model, followed by further structural optimization and dynamics calculations to obtain the final stable structure for representing the simulation model of mycelium-PVA composites (MPCs).

In order to build the molecular dynamics model of MPCs in a water environment, the stable structure of mycelium-assembled PVA was surrounded by water molecules with a thickness of 1 nm and a water molecule mass fraction of 20%. Subsequently, structural optimization and dynamics calculations were carried out to obtain a stable structure of the mycelium-assembled PVA composite in the water environment.

Optimization and dynamics calculations. The initial simulations utilized molecular dynamics (MD) in LAMMPS, employing the polymer consistent force field (pcff) atomic-molecular force field [7]. After 5000 steps of conjugate energy minimization, the system was simulated for 100 ps under constant temperature (300 K) and constant volume conditions. This was followed by 100 ps of equilibration under constant temperature (300 K) and constant pressure (1 bar).

Compressive simulation. The simulation system was held at room temperature and zero pressure was applied in the X, Y and Z directions for a duration of 300 ps. Subsequently, the model was compressed along the Y-axis at a strain rate of $2 \times 10\ 8\ s^{-1}$. To maintain the pressures in the X and Z directions at zero, NPT integral and Nose/Hoover constant pressures were utilized during compression. The system was subjected to periodic boundary conditions in all directions. The atomic stresses in the MD simulations were calculated from the stress tensor in LAMMPS. The stress-strain curves were obtained from the changes in the density and position of the C atoms in the molecule during compressive processing.

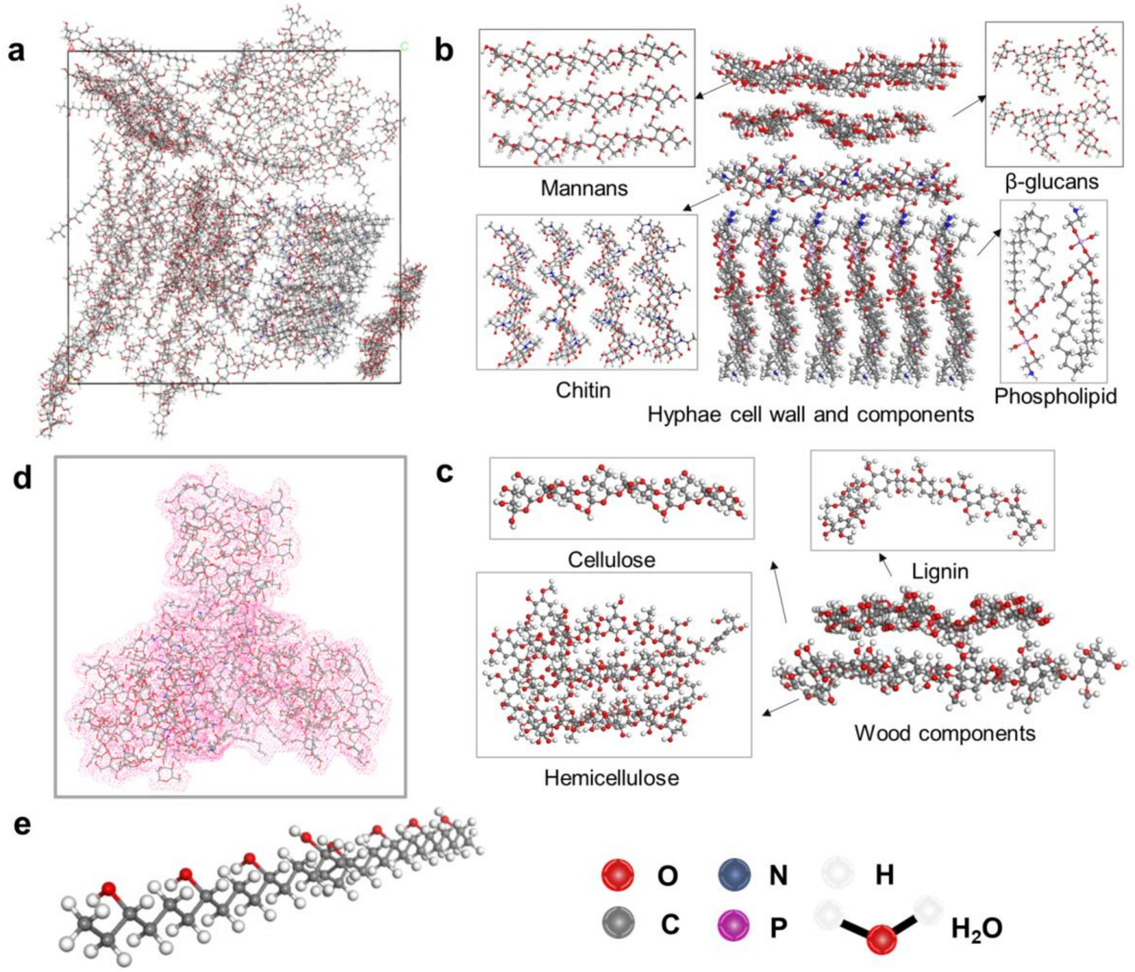

Figure S1 Molecular dynamics (MD) simulation. **a**, The initial model of MPCs. **b**, The hyphae model. **c**, The sawdust model. **d**, The optimized model of the MCs in 10% PVA. **e**, The PVA chain model.

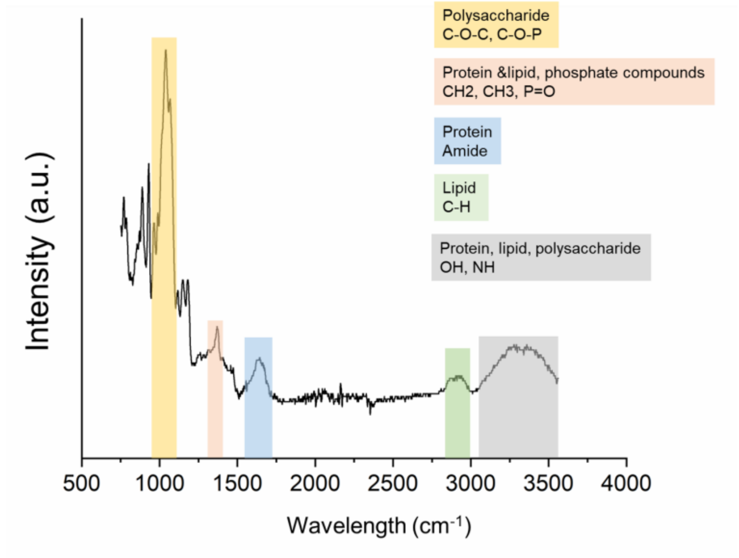

Figure S2 FTIR measurement of the Ganoderma lucidum.

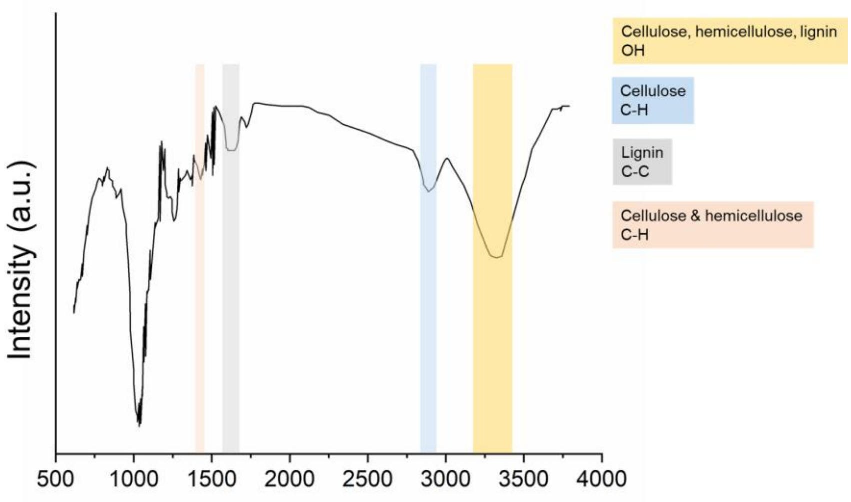

Figure S3 FTIR measurement of the poplar sawdust.

Finite Element Simulation

3D Random Particle Models. Considering the unique spatial structure of the mycelium living composite material, we developed a 3D two-phase mesoscale model to simulate the random meso-structural characteristics of the sawdust and the wrapped mycelium organisms. The mesoscale model was constructed using a 3D random flakes model that incorporates irregular shapes and rough surfaces. **Figure S4** depicts the generation procedure of a 3D random flakes model using the random vector growth method. Firstly, we generated a random quadrilateral *ABCD* inscribed a circle with a diameter of D_a (**Figure S4a**), and the internal angle could be calculated according to the side length as Equation 2. And the robustness of the quadrilateral shape could be controlled by its internal angle and the diameter. Secondly, a random octahedron *EF-ABCD* (**Figure S4b**) was created based on the above-generated quadrilateral *ABCD*, where the vertical distance between points *E* and *F* could be quantitatively controlled using a shape parameter. Thirdly, we have devised a vector-based growth method to generate a randomized polyhedron, building upon the initial octahedron EF-ABCD, as illustrated in **Figure S4c**. The longest edge in the octahedron *EF-ABCD* was selected to determine a random point to be the seed for the following growth procedure. Then an outward vector Vij = Vi + Vj was determined by utilizing the normal vectors (Vi and Vj) of the adjacent planes of the longest edge. Along the vector Vij, a new vertex *G* is then created by randomly adjusting the growth parameter that is related to the spatial coordinates of the newly generated vertex. After connecting all the vertexes, a random decahedron *EF-ABCD* was generated as

presented in **Figure S4d**. Importantly, a correlation exists between the surface count and the duration of random growth. Specifically, after N iterations of random growth starting from a random octahedron, a random polyhedron with a surface count of 2N+8 can be produced.

$$\begin{cases} \text{Cos}\, A = \dfrac{d^2 + c^2 - a^2 - b^2}{2(d \cdot c + a \cdot b)} & \text{(a)} \\ \text{Cos}\, B = \dfrac{d^2 + a^2 - b^2 - c^2}{2(a \cdot d + b \cdot c)} & \text{(b)} \\ \text{Cos}\, C = \dfrac{a^2 + b^2 - c^2 - d^2}{2(a \cdot b + c \cdot d)} & \text{(c)} \\ \text{Cos}\, D = \dfrac{c^2 + d^2 - b^2 - a^2}{2(c \cdot d + a \cdot b)} & \text{(d)} \end{cases} \quad (2)$$

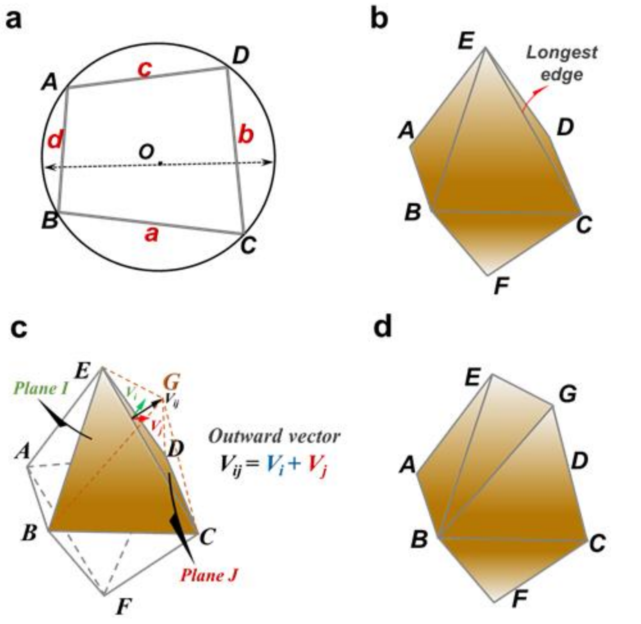

Figure S4 Generation process of a 3D random flakes model. **a**, A random quadrilateral *ABCD* inscribed a circle. **b**, A random octahedron *EF-ABCD* generated from the quadrilateral *ABCD*. **c**, Choosing a random point at the longest edge. **d**, A random decahedron *EF-ABCD*.

As can be seen in **Figure S5a**, random convex flake models with 256 surfaces are generated after 124 times of random vector-growth. Using the "Take & Place" method [8], those established 3D particle models are randomly delivered and distributed in a prescribed specimen domain, as shown in **Figure S5b**. During the delivering process of those random flake models within a spatial domain, the particle overlapping and interaction need to be judged and avoided. If a new particle is found to interact or overlap with the existing one, the spatial coordinates of the former should be adjusted by means of our developed random translation and rotation algorithms, as introduced in Equations 3-4.

$$Rotate\_P_{ri} = Par\_P_i \begin{bmatrix} \cos\beta\cos\gamma & \cos\beta\sin\gamma & -\sin\beta \\ \sin\alpha\sin\beta\cos\gamma - \cos\alpha\sin\gamma & \sin\alpha\sin\beta\sin\gamma + \cos\alpha\cos\gamma & \sin\alpha\cos\beta \\ \cos\alpha\sin\beta\cos\gamma + \sin\alpha\sin\gamma & \cos\alpha\sin\beta\sin\gamma - \sin\alpha\cos\gamma & \cos\alpha\cos\beta \end{bmatrix} \quad (3)$$

$$\begin{cases} \begin{pmatrix} x_{ti} \\ y_{ti} \\ z_{ti} \end{pmatrix} = \chi \begin{pmatrix} r_{11} \\ r_{21} \\ r_{31} \end{pmatrix} + \begin{pmatrix} x_i \\ y_i \\ z_i \end{pmatrix} \\ \left(r_{11}{}^2 + r_{21}{}^2 + r_{31}{}^2\right) \cdot \chi^2 < L_d \end{cases} \quad (4)$$

where α, β and γ respectively represent the rotating angle around x, y and z axes, r_{i1} (i=1, 2, 3) represents the random number list; χ is a translation coefficient, and L_d is a parameter related to the distance relationship between adjacent 3D flake models.

Figure S5b portrays the 3D random packing flake models with a diameter ranging from 3mm to 7mm, representing the spatial stochastic sawdust flakes with irregular shape characteristics. A thin layer with an average thickness of 1-2mm wrapped on the flake surface (marked in white in **Figure S5d-e**), was generated for simulating the mycelium organisms wrapped on the sawdusts. After that, the generated 3D geometry model (**Figure S5e**) was meshed using a 3D mapping meshing method, and a homogenous prismatic model consisting of many hexahedral elements was generated. According to the spatial locations of 3D particle models and the thickness of mycelium organisms layer, the material attribute of each element was ascertained using the generated homogenous finite element model. **Figure S5g** shows the finite element model of a prism specimen of mycelium composites, and the finite element model of those sawdust flakes and the mycelium organisms is respectively presented in **Figure S5c** and **f**. According to the experimental program, the dimensions of the prism specimen is set as 55mm×55mm×30mm. The diameter of sawdust flakes ranges from 3mm to 7mm, and its volume fraction is about 30%. The minimum size of the finite element is set as 0.055mm.

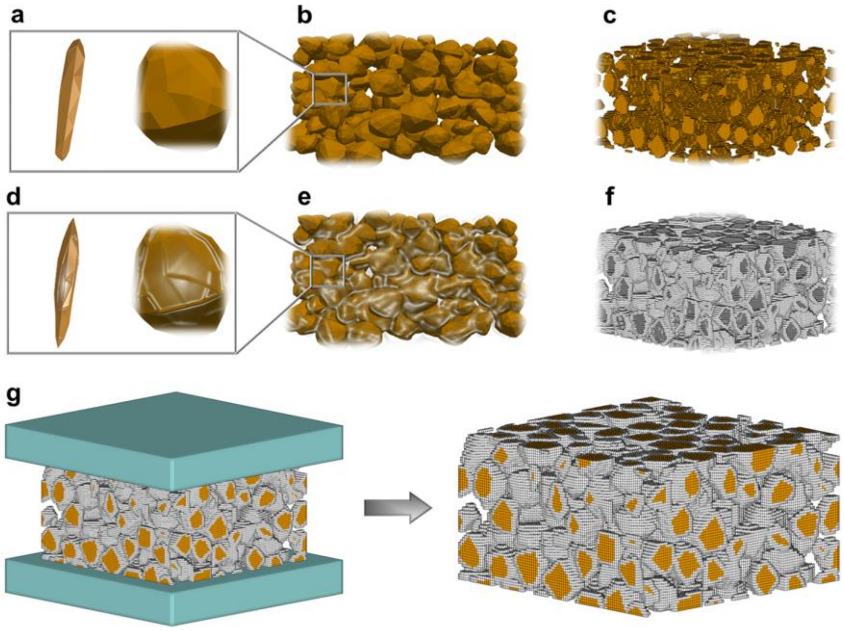

Figure S5 A 3D two-phase mesoscale model for the MPCs. **a** and **d** show the 3D convex flakes model with random shape in angular feature and rough surface, which is used for modelling the sawdust. While the white phase appeared on the particle surface represents the sawdust wrapped with the mycelium organisms. Similarly, **b**, exhibits the 3D random packing model of sawdusts, while **e** is the 3D packing model wrapped with the mycelium organisms. **c**, **f**, **g** presents the finite element model of sawdusts, mycelium organisms and the MPCs, respectively.

Finite element calculation. Using the commercial software such as ANSYS and LS-PREPOST [9], the finite element calculation of the MPCs subjected to axial compression load are performed. It should be pointed out that the *Mat_Wood (*Mat_143) model and the *Mat_Fabric (*Mat_034) model in LS-DYNA, is selected to simulate the material behaviors of the sawdust material and the mycelium organism, respectively. The basic material parameters of sawdust material are listed as follows: mass density ρ=0.35g/cm^3, Young's modulus in axial direction Ea=11GPa, Young's modulus in transversal direction Ea=0.37GPa, shear modulus G=0.69GPa, Poisson's ratio μ=0.4, shear strength 2.5MPa, compressive strength fc=20MPa, tensile strength ft=2Mpa. The basic analytical parameters of chitin component of mycelium organism are: ρ=1.43g/cm^3, Young's modulus E=2.5GPa, μ=0.3, ft=1Mpa. The axial compression load is controlled by the displacement applied to the upper steel plate (see **Figure S5g**), and the bottom steel plate was constrained at XYZ directions. The loading rate was set as 1mm/s. We use the keyword of *Automatic_Surface_To_Surface in LS-DYNA to simulate the contact behavior between sawdusts and mycelium organism.

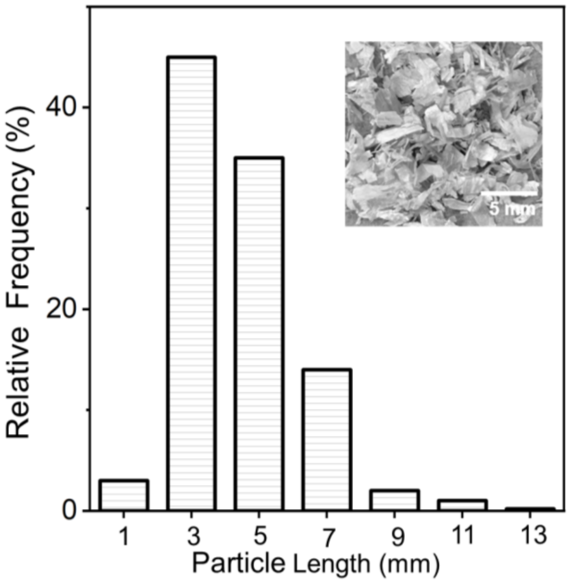

Figure S6 Relative length frequencies of sawdust showing that the size range of sawdust is 3-7 mm.

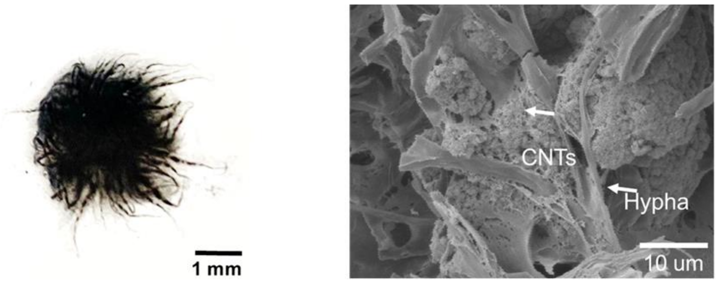

Figure S7 MCs in 5% CNTs after growth 3 days. Living MCs are employed to induce phase separation in CNTs suspensions, resulting in the aggregation of CNTs that form a coating on the hyphae surface.

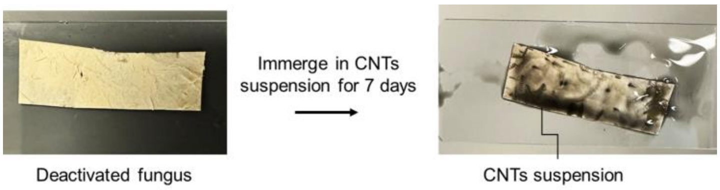

Figure S8 Inactivated fungi are placed in nutrient solution containing CNTs for 7 days.
In the experimental setup, the fungi, which are rendered inactive or inactivate, are immersed in a nutrient solution containing 5% CNTs for a period of 7 days. The observation indicates that the CNTs suspension does not undergo phase separation on the surface of the mycelium. This suggests that, during the specified duration, the CNTs did not separate or form distinct phases on the mycelium surface in the given conditions.

Table S1 Physico-mechanical properties of naturally grown mycelium bio composite materials

Material	Density (g/cm^3)	Strength (MPa)	Specific Strength ($MPa/g.cm^{-3}$)	Reference
Mycelium -wheat straw	0.192	0.17	0.89	[10]
Mycelium- sawdust	0.13-0.552	up to 1.1	1.6-1.99	[10,20]
Mycelium- biodegradable agro waste particle	0.121-0.133	up to 0.05	0.33-0.38	[11,12]
Mycelium-rice straw	0.265	0.299-0.304	1.13-1.15	[13]
Mycelium-bagasse	0.304	up to 0.35	1.12-1.14	[14]
Mycelium coir-pith	0.298	up to 0.35	1.14-1.16	[15]
Mycelium-corn straw	0.249	up to 0.299	1.08-1.2	[16]
Mycelium-cotton carpel, cotton seed hull, starch, gypsum	0.067–0.224	up to 0.24	0.32-1.07	[17]
Mycelium-wood chips, hemp hurd, loose hemp fiber	0.170–0.260	up to 0.143	0.36-0.55	[20]
Mycelium-dried branches, ground woodchips, flour, wheat straw	0.174–0.245	up to 0.52	0.45-2.12	[18]
Mycelium-sawdust pulp of Alaska birchmillet grain, wheat bran, calcium sulfate	0.169–0.280	up to 0.94	1.25-3.37	[19]
Mycelium-bacterial cellulose	0.98-1.2	up to 1.7 (Tensile test)	1.16-1.42 (Tensile test)	[20]
Mycelium-cellulose	0.16-0.28	up to 0.43	0.89-1.54	[21]
EPS	0.012-0.029	up to 0.38	1.21-13.16	[22]
MPCs	0.27	up to 2.2MPa	7.6-8.15	This work

Table S2 Water absorption of mycelium bio composite materials

Material	Density(g/cm^{-3})	Water absorption weight ratio	Reference
Mycelium-wood, cellulose nanofibrils composites	0.16-0.28	220%-245% (24 hours)	[23]
Mycelium-rapeseed straw	0.1-0.13	363%-509% (72 hous)	[24]
Mycelium-cotton	0.13	432%-584% (72 hous)	[24]
Mycelium-yellow birch wood	0.17	150%-280% (72 hous)	[25]
MPCs	0.274	33% (15 days)	This work

References

[1] Girometta, C. et al. Characterization of mycelia from wood-decay species by TGA and IR spectroscopy. Cellulose 27(11), 6133–6148 (2020).

[2] Liu Y.N., Wu Q., Wu X.Y., Algharib S.A., et al. Structure, preparation, modification, and bioactivities of β-glucan and mannan from yeast cell wall: A review. International Journal of Biological Macromolecules, 173,, 445-456 (2021).

[3] Vega K., Kalkum M. Chitin, Chitinase Responses, and Invasive Fungal Infections. International Journal of Microbiology, (1): 920459 (2012).

[4] Gow N.A.R., Lenardon M.D. Architecture of the dynamic fungal cell wall. Nat. Rev. Microbiol., 21, 248–259 (2023).

[5] Gu X.L., Ma X., Li L.X., et al. Pyrolysis of poplar wood sawdust by TG-FTIR and Py–GC/MS. Journal of Analytical and Applied Pyrolysis, 102, 16-23 (2013).

[6] Wang H.M., Ma C.Y., Li H.Y., et al. Structural Variations of Lignin Macromolecules from Early Growth Stages of Poplar Cell Walls. ACS Sustainable Chem Eng, 8 (4), 1813-1822 (2020).

[7] Yungerman I., Starodumov I., Fulati A., et al. Full-atomistic optimized potentials for liquid simulations and polymer consistent force field models for biocompatible shape memory poly(ε-caprolactone). J. Phys. Chem. B., 126, 3961−3972. (2022).

[8] Wriggers P., Moftah S. O. Mesoscale models for concrete: Homogenisation and damage behaviour. Finite elements in analysis and design, 42(7), 623-636 (2006).

[9] L.S.T.C. LS-DYNA version 971 keyword user's manual. Livermore Software Technology Corporation, California, USA (2007).

[10] Elsacker E., Vandelook S., Brancart J., et al. Mechanical, physical and chemical characterisation of mycelium-based composites with different types of lignocellulosic substrates. PLoS ONE 14(7): e0213954 (2019).

[11] Pohl C., Schmidt B., Nunez Guitar T. et al. Establishment of the basidiomycete Fomes fomentarius for the production of composite materials. Fungal. Biol. Biotechnol., 9, 4 (2022).

[12] Jones M., Mautner A., Luenco S., et al. Engineered mycelium composite construction materials from fungal biorefineries: A critical review. Mater. & Des., 187 (2020).

[13] Peng L.C., Yi J., Yang X.Y., et al. Development and characterization of mycelium bio-composites by utilization of different agricultural residual byproducts. J. Bioresour. & Bioprod., 8 (1), 78-89 (2023).

[14] Islam M.R., Tudryn G., Bucinell R. et al. Mechanical behavior of mycelium-based particulate composites. J. Mater. Sci., 53, 16371–16382 (2018).

[15] Scott W., Huynh T., John S. Hybridizations and reinforcements in mycelium composites: A review. Bioresource Technology Reports, 22, ,101456 (2023).

[16] Rigobello A., Ayres P. Compressive behaviour of anisotropic mycelium-based composites. Sci. Rep., 12, 6846 (2022).

[17] Pelletier M, Holt G, Wanjura J, et al. Acoustic evaluation of mycological biopolymer, an all-natural closed cell foam alternative. Ind. Crops Prod., 139, 111533 (2019).

[18] Attias N., Danai O., Abitbol T., Tarazi E., Ezov N., et al. Mycelium bio-composites in industrial design and architecture: comparative review and experimental analysis. J. Cleaner. Prod., 246, 119037 (2020).

[19] Yang Z., Zhang F., Still B., White M., Amstislavski P. Physical and mechanical properties of fungal mycelium-based biofoam. J. Mater. Civ. Eng., 29 (7), 04017030, (2017).
[20] Elsacker E., Vandelook S., Damsin B. et al. Mechanical characteristics of bacterial cellulose-reinforced mycelium composite materials. Fungal. Biol. Biotechnol., 8, 18 (2021).
[21] Davine Blauwhoff I.L.B.From Biomass to Mycelium Composite an Exploration on Cellulose and Weed Residues, Stowa (2019).
[22] Gou L.Y., Li S., Yin J.S., Li T.T., Liu X. Morphological and physico-mechanical properties of mycelium biocomposites with natural reinforcement particles. Construc. & Build. Mater., 304,124656 (2021).
[23] Sun W., Tajvidi M., Hunt C.G. et al. Fully Bio-Based Hybrid Composites Made of Wood, Fungal Mycelium and Cellulose Nanofibrils. Sci. Rep., 9, 3766 (2019).
[24] Appels Freek V.W., Camere S., Montalti M., et al. Fabrication factors influencing mechanical, moisture- and water-related properties of mycelium-based composites. Mater. & Des.,161, 64-71(2019).
[25] Sun W.J., Tajvidi M., Howell C., Hunt C.G., Insight into mycelium-lignocellulosic bio-composites: Essential factors and properties, Compos. Part A: Appl. Sci. & Manuf., 161,107125 (2022).
